# Reply to Hayashi and Konishi: Noneffect of SARS-CoV-2 spike glycoprotein Y217N mutation on affinity between virus and ACE2

**DOI:** 10.1016/j.jbc.2021.100706

**Published:** 2021-05-31

**Authors:** Yan-Dong Tang

**Affiliations:** State Key Laboratory of Veterinary Biotechnology, Harbin Veterinary Research Institute of the Chinese Academy of Agricultural Sciences, Harbin, China

We thank Hayashi and Konishi for their comments and interest in our article (1). We agree that by structural modeling analysis, angiotensin-converting enzyme 2 (ACE2 [Y217N]) mutant shows no changes in binding with receptor-binding domain (RBD) when compared with WT ACE2, which is depicted in Figure 4 in our article. We think the lower binding affinity of human ACE2 N217 with RBD was due to the following reasons. First, cell surface abundance of ACE2 Y217N was less compared with WT ACE2 (Fig. 5 in our article). Second, although cell surface abundance of ACE2 Y217N decreased, there were more than 20% of cells with ACE2 positive. Nevertheless, when quantified by Western blot, RBD binding was extremely low (Fig. 3 in our article). Therefore, we think human ACE2 N217 lost the binding ability to RBD and speculated that introducing Y217N mutation may alter the folding and conformational structure of ACE2. However, this needs to be investigated further.

## Conflict of interest

The author declares no conflicts of interests with the contents of this article.
